# Occipital Proton Magnetic Resonance Spectroscopy (^1^H-MRS) Reveals Normal Metabolite Concentrations in Retinal Visual Field Defects

**DOI:** 10.1371/journal.pone.0000222

**Published:** 2007-02-21

**Authors:** Christine C. Boucard, Johannes M. Hoogduin, Jeroen van der Grond, Frans W. Cornelissen

**Affiliations:** 1 BCN Neuro-imaging Center, University Medical Center Groningen, University of Groningen, Groningen, The Netherlands; 2 Laboratory for Experimental Ophthalmology, University Medical Center Groningen, University of Groningen, Groningen, The Netherlands; 3 Department of Radiology, University Medical Center Leiden, Leiden, The Netherlands; University of Southern California, United States of America

## Abstract

**Background:**

Progressive visual field defects, such as age-related macular degeneration and glaucoma, prevent normal stimulation of visual cortex. We investigated whether in the case of visual field defects, concentrations of metabolites such as N-acetylaspartate (NAA), a marker for degenerative processes, are reduced in the occipital brain region.

**Methodology/Principal Findings:**

Participants known with glaucoma, age-related macular degeneration (the two leading causes of visual impairment in the developed world), and controls were examined by proton MR spectroscopic (^1^H-MRS) imaging. Absolute NAA, Creatine and Choline concentrations were derived from a single-voxel in the occipital region of each brain hemisphere. No significant differences in metabolites concentrations were found between the three groups.

**Conclusions/Significance:**

We conclude that progressive retinal visual field defects do not affect metabolite concentration in visual brain areas suggesting that there is no ongoing occipital degeneration. We discuss the possibility that metabolite change is too slow to be detectable.

## Introduction

The two leading causes of visual impairment in the developed world, age-related macular degeneration (AMD) and glaucoma [1], are affected by progressive retinal visual field defects. Due to the retinotopic cortical organisation, when these field defects occur in both eyes and overlap, a corresponding section of visual cortex no longer receives stimulation. It is known that prolonged absence of stimulation may result in cortical changes [2], [3]. In this paper, we studied if the concentrations in the occipital brain of the metabolites N-acetylaspartate (NAA) (a marker of neuronal integrity), Creatine (Cr) and Choline (Cho) are affected by progressive visual field defects. Absolute concentrations of these compounds were measured in the occipital region using single-voxel proton magnetic resonance spectroscopy (^1^H-MRS) in each hemisphere of AMD and glaucoma patients and a control group.

^1^H-MRS is a non-invasive technique that allows detection and quantification of certain biochemical compounds in brain tissue, such as NAA, Cr, Cho, and lipids [4], [5]. NAA is found at relatively high concentrations in the human central nervous system and is particularly localized within neurons and related to neuronal processes [4], [6]. A decrease in its concentration is routinely considered as an indicator of neuronal loss or dysfunction [7], [8] and has been observed in different brain regions in various neurodegenerative disorders [5], [9]–[13] and neuro-ophthalmology [14]. Its decrease is mostly observed at the moment when the disease is in progression. Cr, which is known to play an important role in energy metabolism, has been reported to be constant throughout the brain and resistant to change in several degenerative brain diseases [4], [15], [16]. Cho is considered a marker for cell turnover [4].

AMD is caused by the accumulation of waste products in the tissues underneath the macula preventing normal retinal metabolism and leading to gradual retinal atrophy [17], [18]. AMD affects the retinal pigment epithelium and the photoreceptor layer, and causes centrally located visual field defects. In glaucoma, visual field loss starts peripherally. Progressive retinal ganglion cell loss and optic nerve damage occur, in most cases, induced by an elevated intra-ocular pressure [19], [20]. In both cases, the retinal damage is in continuous progress.

NAA is considered a major marker for neuronal integrity. If progressive visual deprivation affects the metabolism of the adult visual brain, lower local NAA concentrations in the occipital areas are expected for the visual field defect groups when compared to the control group. Our results, however, indicate no significant differences in metabolite concentrations among the visual field defect and the control groups. This can be interpreted as degeneration not currently occurring or occurring at a very slow rate preventing detectable changes.

## Materials and Methods

### Subjects

Subjects with visual field defects were recruited from a database of the Department of Ophthalmology of the University Medical Center Groningen (Groningen, The Netherlands) and through advertisements in magazines of patient associations. The group consisted of seven patients suffering from AMD (two female and five males; mean age 72 years, range 52–82) and seven patients with primary open-angle glaucoma (one female and six males; mean age 73 years, range 61–84). Patients had to have, for a minimum of 3 years, homonymous scotoma of at least 10 degrees diameter located centrally in at least one quadrant (as recorded using the 30-2 program Sita Fast of Humphrey Field Analyzer (Carl Zeiss Meditec, Dublin, California, USA)). Patients with any other (neuro-) ophthalmic disease that might affect the visual field were excluded.

For the control group, 12 healthy subjects (four female and eight male; mean age 62 years, range 46–82) were recruited either by advertisement in a local newspaper, or were the partners of the visual field impaired participants. Control subjects were required to have good visual acuity, not to have any visual field defect, and had to be free of any ophthalmic, neurologic, or general health problem.

This study conformed to the tenets of the Declaration of Helsinki and was approved by the medical review board of the University Medical Center Groningen (Groningen, The Netherlands). All participants gave their informed written consent prior to participation.

### Materials and data acquisition

**Figure 1:**
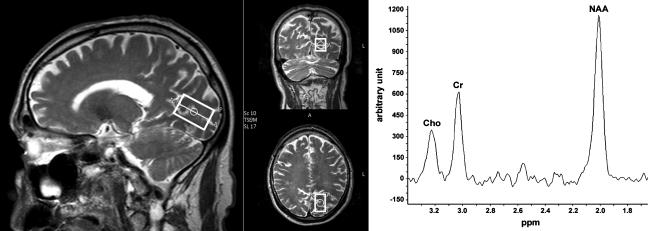
Example of PRESS box position and spectra. *Left*: To delineate the single-voxel, an elongated PRESS box was located in each hemisphere (here shown only in the left hemisphere) along the calcarine sulcus as far to the back of the occipital pole and the midline of the brain as possible while avoiding the inclusion of fat and vasculature. *Right*: Example of spectra with Choline, Creatine and NAA peaks.

Single-voxel ^1^H-MRS was performed on a whole body 3.0 Tesla Philips Intera scanner (Eindhoven, The Netherlands) in both the left and right occipital pole using the standard T/R headcoil. Scanning parameters were: TE = 144ms, TR = 2s and 128 signal averages. To delineate the single-voxel or region of interest (ROI), an elongated PRESS box was located in each hemispheres along the calcarine sulcus as far to the back of the occipital pole and the midline of the brain as possible while avoiding the inclusion of fat and vasculature (figure 1). Total scan time was 5 min per ^1^H-MRS voxel including acquisition of an unsuppressed water signal with identical scanner settings.

Raw signals were post processed using the scanner software. Post-processing included: 1) DC baseline correction using the last 10% of the signal. 2) Multiplication with a Gaussian and exponential function resulting in 2 Hz line broadening and 1 Hz line sharpening, respectively. 3) Zero filling from 1024 to 4096 samples. 4) Fourier transformation from the time to the frequency domain. 5) Manual zero and first order phase correction.

Baseline and peak heights were determined manually by 3 independent operators, naïve with respect to the subject's classification. The measurements showed high correlation between operators and were averaged.

Absolute metabolite concentrations were obtained by using the unsuppressed water spectrum as a reference and assuming a water volume percentage of 71%.

### Statistics

Because no significant differences in metabolite concentrations between the right and left hemispheres were detected, these values were averaged. A one-way analysis of variance (ANOVA) was used to determine any significant difference in the concentration of the three metabolites (NAA, Cr and Cho) between the three groups (AMD, glaucoma and controls).

## Results

**Figure 2:**
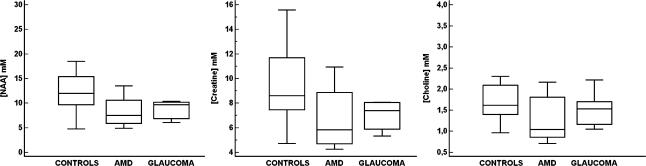
Metabolite concentrations. Averaged absolute concentrations (in nM) of each of the measured metabolites (NAA, Cr and Cho) for each group (controls, AMD and glaucoma). The ANOVA analysis between the three groups (AMD, glaucoma and controls) showed no significant differences for any of the three metabolites concentration.

Boxplots in figure 2 depict the averaged absolute concentrations of each of the measured metabolites (NAA, Cr and Cho) for each group (controls, AMD and glaucoma).

The one-way ANOVA between the three groups (AMD, glaucoma and controls) showed no significant differences for any of the three metabolites concentration. For the NAA concentrations: F(2,23) = 2.433, p<0.110; for the Cr concentrations: F(2,23) = 2.144, p<0.140; and for the Cho concentrations: F(2,23) = 1.754, p<0.195.

## Discussion

The main finding of this study is that, the NAA absolute levels in the occipital brain of subjects with progressive visual field defects (AMD and glaucoma) do not differ from the levels of a group of control subjects. Hence, our results indicate that progressive retinal visual field defects do not induce a measurable decrease in NAA metabolite concentration in the visual brain areas.

AMD and glaucoma are both accompanied by progressive visual field defects. As a consequence of that and because of the retinotopic organisation of visual cortex, the section of cortex corresponding to the dysfunctional area of the retina will no longer receive input. Indeed, previous work by our group (submitted for publication) showed that compared to controls, glaucoma, but not AMD, patients showed a lower grey matter (GM) concentration as a result of cortical thinning in the cortical lesion projection zone. Knowing that non-stimulated cortical tissue tends to degenerate [2] and that occurring cell loss is linked to a decrease in NAA metabolite concentration [7], [8], [21], we would have expected to find a reduction in NAA concentration in the visual brain of patients suffering from progressive visual field defects, in particular those with glaucoma. However, this is not the case. The fact that in our sample there was no significant lower level of NAA suggests that cell loss is not currently occurring in the occipital brain of our patient groups. Longitudinal studies have emphasized that a substantial proportion of the decreases in NAA occurs in the acute phase of cell degeneration [21].

Visual field degeneration in both AMD and glaucoma progresses rather slowly [22]. This fact suggests that perhaps the rate of progression is not high enough to evoke a decrease in NAA metabolite concentration. Alternatively, the cortical area corresponding to the affected retinal region may be too small to provoke NAA changes that can be measured using single-voxel ^1^H-MRS.

On the other hand, ROI defined by our single-voxel may not completely cover a degenerated region. In a previous magnetic resonance imaging study (submitted for publication) we investigated changes in GM. The area where differences were detected did correspond to the projection zone of the damaged region of the retina and was located in the anterior occipital lobe, along the interhemispheric fissure. Because of its proximity to the fissure, and consequently its vicinity to large amounts of pulsating blood, this area is unsuitable for a single-voxel ^1^H-MRS ROI. As a consequence, our present ROI locations may not have been placed exactly in the cortical region previously associated with a reduction in GM. This may have reduced our ability to demonstrate small local changes in metabolite concentration.

Our results show no significant differences in Cr concentrations between all three groups, as well. This was expected since this metabolite has been observed to stay invariable throughout the brain, also in the case of degenerative brain disorders [4], [15], [16]. Therefore, normalized changes in NAA are often assessed in relation to Cr in terms of the ratio NAA/Cr [23]. However, despite being resistant to change in degenerative diseases, we preferred not to make use of the ratio NAA/Cr to measure cell loss. Variations in Cr levels do occur as general loss together with other metabolites in tissue necrosis [4]. Thus, if the process of degeneration has already taken place and has stopped, NAA/Cr levels will not decrease but both NAA and Cr compounds will be comparably reduced as a result of the decay of cell number. The ratio NAA/Cr will consequently be inadequate to evaluate any changes in NAA.

Increases in Cho levels have been related to cell turnover [4]. Again, the fact that in our experiment no changes were measured in Cho levels among all three groups reveals that no cortical metabolic changes are associated with visual field defects.

With the relatively small size of our experimental groups, the present study has only the power to detect big effects. This is not necessarily a disadvantage, because relatively large effects would have had more potential clinical implication than subtle differences. On the other hand, larger sample sizes could allow identification of more subtle differences, the presence of which is suggested by trends in our analysis.

Finally, AMD and glaucoma are both associated with the occurrence of visual field defects but differ in the pathology. In glaucoma, the optic nerve is damaged, while in AMD it remains intact. We thus observe that nerve damage does not necessarily affect NAA levels in the occipital region. Conversely, an immunohistochemistry study targeting the mechanism behind multiple sclerosis linked artificially induced optic nerve damage in the rat to a decrease in NAA concentration, which returned to normal level after 24 days [24]. Likewise, another group measured a lower NAA concentration in the chiasm of two patients suffering from optic neuritis. After visual field improvements, the NAA levels increased [25]. This is in agreement with the idea that reduced NAA concentrations can only be measured when degenerative processes are currently taking place. In the case of our patient groups, the absence of a detectable change suggests either that occipital degeneration had already occurred (as presumably is the case of glaucoma) or perhaps occurs at a rate that is too slow to induce detectable changes in NAA concentrations.

In conclusion, no significant differences in NAA, Cr and Cho absolute concentrations were found in the occipital brain of patients suffering from visual field defects when compared with controls. The absence of a reduction in NAA concentrations compared to controls most likely indicates that no degeneration is currently occurring in the occipital region of AMD and glaucoma patients. This absence might also be due to the fact that both diseases progress at a very slow rate, which may prevent detectable NAA changes. Further research concerning ocular disorders with a faster degenerative process (for instance, as in ischemic optic neuropathy or retinal vascular occlusion) could clarify this issue. The application of ^1^H-MRS for the metabolic evaluation of consequences of retinal visual field defects in the visual brain may help understand disease symptoms and progression as well as the mechanisms of brain plasticity in general.
